# Effect of Cleaning Protocols on Surface Roughness of Current Polymeric Denture Materials

**DOI:** 10.3390/jfb16100359

**Published:** 2025-09-24

**Authors:** Lisa Brinkmann, Florian Fuchs, Martin Rosentritt, Oliver Schierz, Andreas Koenig, Daniel R. Reissmann

**Affiliations:** 1 Department of Prosthodontics and Materials Science, Leipzig University, Liebigstraße 12, 04103 Leipzig, Germanydaniel.reissmann@medizin.uni-leipzig.de (D.R.R.); 2 Department of Prosthetic Dentistry, Regensburg University Medical Center, 93042 Regensburg, Germany; 3 Department of Prosthodontics and Materials Science, Rostock University, Strempelstraße 13, 18057 Rostock, Germany; oliver.schierz@med.uni-rostock.de

**Keywords:** CAD/CAM, toothbrush, abrasion, confocal scanning laser microscopy, surface texture analysis, ISO 25178, Vickers hardness, Martens hardness, polymer

## Abstract

Surface roughness influences biofilm adhesion on denture base materials, impacting oral health. Despite advances in polymeric denture materials, the effects of common cleaning protocols on their surface texture remain inadequately characterized. This study investigated the influence of toothbrush abrasion on the surface texture of dimethyl methacrylate-based (DMA, printed: V-Print dentbase), polymethyl methacrylate (PMMA, milled: VITA Vionic Base, pressed: IvoBase Hybrid), polyamide (PA, pressed: Bre.flex), and polyether ether ketone (PEEK, milled: Juvora Disc). The specimens were fabricated as polished discs. The Vickers and Martens hardness, indentation modulus, elastic and plastic part of indentation work, and indentation creep were determined. Toothbrushing simulation and surface texture analysis were conducted in three steps: 1800, 1800, and 3600 cycles using water, dish detergent, or toothpaste slurry. The surface texture parameters Sa, Sal, Sdr, Sku, and Ssk were determined using confocal laser scanning microscopy and suitable filtering (S-F and S-L surface). Sa, Sal, and Sdr showed significant changes depending on the choice of medium and the material used. The duration had a small effect (three-way ANOVA; all *p* < 0.001). DMA showed minor surface changes. Milled and pressed PMMA exhibited similar surface deformities due to wide valleys that were not considered critical for biofilm adhesion. PA showed the lowest and PEEK the highest Vickers and Martens hardness. However, both PA and PEEK exhibited surface changes that could promote biofilm development. These findings suggest that denture cleaning recommendations should remain material-specific. Regular surface inspections and repolishing are necessary to reduce the risk of biofilm formation on PA or PEEK-containing dentures.

## 1. Introduction

Removable dentures are a therapeutic option for tooth loss to restore function, aesthetics, and oral health-related quality of life [1]. In Germany, 29.9% of young senior citizens (65–74 years) wear removable dentures [2]. Digitalization has brought new materials and manufacturing technologies for dentures to the fore. Important representatives include polymethyl methacrylate (PMMA), polyether ether ketone (PEEK), and polyamide (PA) for thermoplastics and dimethyl methacrylate (DMA)-based polymers for thermosets. The manufacturing process and material are not entirely selectable, as they depend on the polymerization and polymer properties. PMMA remains the most common denture base material [3]. It is manufactured by thermoplastic pressing or milling from industrially polymerized blanks. Many comparative studies have been carried out on the different manufacturing methods. Thus, industrially polymerized PMMA has been shown to have a lower residual monomer content [4] and improved mechanical properties [5]. Furthermore, there are indications that microorganisms accumulate less on industrially polymerized than on pressed-produced PMMA [6]. DMA-based polymers are well-known in the field of composites and are now being used as resins via 3D printing in the manufacturing of digitally fabricated dentures. An increased residual monomer content for printable materials should be avoided by sufficient post-polymerization [7]. PEEK and PA are alternative base materials for material intolerance (e.g., with MMA) [8,9,10]. Manufacturing is predominantly carried out by milling for PEEK and pressing for PA [11]. Other options, such as 3D printing, are not yet fully developed [12].

The higher ductility and lower hardness as well strength of acrylics compared to composites or ceramics means that, in addition to mechanical failure due to masticatory forces, mechanical resistance to abrasive stresses (tooth brushing with/without additives, food) is also decisive for their use as a denture base [13]. Similar to DMA-based polymers, PEEK is known for its improved mechanical properties compared to PMMA (e.g., PEEK/DMA/PMMA: flexural strength 190–210/51–69/118–146 MPa; modulus of elasticity: 4.2–4.8/3.0–3.1/2.5–4.9 GPa; and Vickers hardness 21–27/21–25/19–22 HV [14,15,16,17,18,19,20]) and higher abrasion resistance (material loss after 120,000 mastication cycles 0.77/0.69/0.89 mm^3^) [21,22]. Compared to PEEK, PMMA, and DMA, PA has the lowest mechanical performance (flexural strength 70 MPa/Vickers hardness 8.0) and is characterized by a very high toughness (group: soft-elastic plastic) [23,24]. When determining the universal hardness (also known as Martens hardness), further mechanical parameters can be determined that enable an assessment of the elastic/plastic load–deformation behavior. These include the load–deformation ratio in the linear-elastic range (indentation modulus, E_IT_), the load-dependent long-term deformation behavior (indentation creep, C_IT_), and the plastic and elastic mechanical energy expenditure (mechanical work, W_plast_, W_elast_) [25,26,27].

Surface roughness is a major factor in the adhesion of biofilm to dental materials. Depending on the patient’s immune status and health condition, microorganisms can have an impact on general health through denture stomatitis and aspiration pneumonia [28]. To date, no generally valid cleaning recommendation for dentures has been established [29,30]. Damage to the surface of dental prosthetic materials caused by mechanical cleaning must be considered [31,32,33]. Even if non-abrasive cleaning agents such as rinsing agents are recommended, cleaning with toothpaste, which is an integral part of oral hygiene for many patients, should be taken into account [29,34]. Possible damage to the surface can be analyzed using 3D surface texture analysis. In addition to the widely used parameter of the arithmetic mean height (Sa), further parameters can provide information about the distribution of the height (kurtosis, Sku; skewness, Ssk), the available surface area (developed interfacial area ratio, Sdr), or the size of particular surface features (auto-correlation length, Sal). A suitable filtering of the coarse form (F-operator), and of small (S-filter) as well as large (L-filter) surface features allows more precise analyses in different orders of scale [35]. The resulting S-F (or S-L) surfaces represent quantifiable surfaces for large (or small) surface features, which were formerly referred to as “waviness” (or “roughness”) in line/profile measurements. Even though the terms waviness/roughness allow for a more intuitive description, the correct method description should be made according to ISO 25178 [35,36,37].

**Figure 1:**
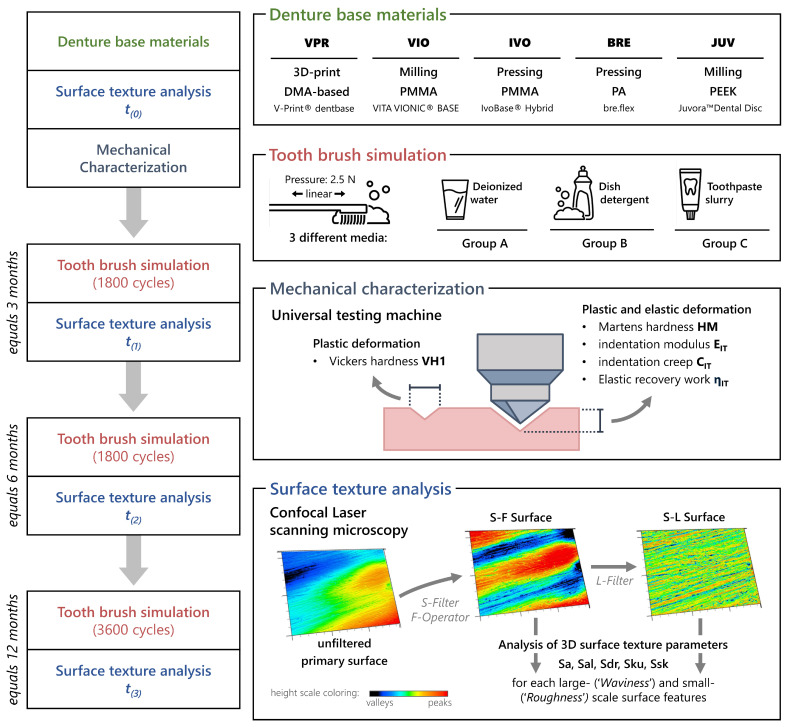
Study design with corresponding representation for the artificial aging and analysis of the test specimens.

The aim of this study was a comprehensive analysis of the 3D surface texture of current denture base materials using various cleaning protocols by means of toothbrushing simulation (Figure 1). The null hypothesis was that the toothbrushing simulation and the factors medium (water, dish detergent, toothpaste slurry), material (VPR, VIO, IVO, BRE, JUV), and the number of cycles (t_(0)_, t_(1)_, t_(2)_, t_(3)_) have no effect on the parameters Sa, Sal, Sdr, Sku, and Ssk.

In addition, mechanical tests enabled a supplementary characterization of the materials. The research hypothesis was that harder materials exhibit smaller changes in the surface texture due to mechanical loading.

## 2. Materials and Methods

### 2.1. Sample Preparation

A total of 60 test specimens were produced from the following denture base materials: V-Print dentbase (VPR), Vita Vionic Base (VIO), IvoBase Hybrid (IVO), Bre.flex 2nd Edition (BRE), and Juvora Disc (JUV) (see Table 1). All materials were produced as cylinders (Ø: 12 mm) according to the manufacturer’s instructions. For IVO/BRE, the cylinders were manufactured using a conventional compression molding technique. VPR was printed as a cylinder at a 10° angle. All cylinders were then cut into slices with 2.5 mm thickness using a precision saw (IsoMet 4000, Buehler Ltd., Lake Bluff, IL, USA). Using a semi-automatic polishing machine (Pedemin-II/DAP-V, Struers, Ballerup, Denmark), uniform surface finishes were produced by grinding with SiC paper (up to P4000) and subsequent polishing via 3 µm diamond suspension (STRUERS DAC). The resulting specimens were then ultrasonically cleaned with deionized water for 5 min, dried with compressed air, and divided into three groups.

### 2.2. Mechanical Characterization

To interpret the material-specific surface changes, the load–indentation deformation according to ISO 14577-1 and the Vickers hardness based on DIN EN ISO 6507-1 were determined on three polished specimens at a total of 18 points per material using the ZHU/zwickiLine Z2.5 universal hardness testing machine (ZwickRoell, Ulm, Germany) [38,39]. The loading and unloading speed was 2 N/s, with a holding time of 14 s and a maximum force of 10 N. The specimens had an equilibrium moisture content and were tested at a standard climate of 20 °C using the “testXpert Version 12.6” software. The following mechanical parameters were considered in this study: the Vickers hardness (HV1), the Martens hardness (HM), the indentation modulus of elasticity (E_IT_), the elastic and plastic part of indentation work (W_elast_ and W_plast_), and the creep factor (C_IT_).

### 2.3. Toothbrushing Simulation

The test specimens were allocated to one medium each (group A, B, or C) and a toothbrushing simulation (ZM-3, SD Mechatronik, Feldkirchen-Westerham, Germany) with linear brushing movements, a contact pressure of 2.5 N, and a toothbrush (elmex short-head toothbrush, flat bristle field, medium hardness). Group A was brushed with distilled water and Group B with a 50:50 mixture of dish detergent (Domol ultra sensitive, Dirk Rossmann GmbH, Burgwedel, Germany) and distilled water. For group C, a toothpaste slurry of 250 g toothpaste (Colgate Total, Colgate-Palmolive Company, Warsaw, Poland; RDA: 78) and 1000 mL distilled water was prepared. To simulate different time points, the test specimens were subjected to toothbrushing simulation for 1800 cycles (t_(1)_; equals 3 months), an additional 1800 cycles (t_(2)_; equals 6 months), and a further 3600 cycles (t_(3)_; equals 12 months). The test specimens were analyzed before (baseline, t_(0)_) and after (t_(1)_, t_(2)_, t_(3)_) the described time intervals using confocal laser scanning microscopy (VK-X1000/X1050, Keyence, Osaka, Japan; λ = 661 nm; detector resolution: 2048 × 1536 px) at 50× magnification (CF IC EPI Plan 50x, N = 0.8; WD = 0.54 mm, Nikon, Tokio, Japan). To ensure a uniform surface texture, the same orientation of the test specimens was maintained over the different time intervals by marking.

### 2.4. Surface Texture Analysis

For the qualitative assessment of the surface, the specimens were documented using macro photography under constant illumination with the following setup: camera (OM-D EM-1 Mark II, OLYMPUS, Tokyo, Japan; 50 MP), lens (MP-E 65 mm f/2. 8 1–5x, CANON, Tokyo, Japan; AES-MFT COMMLITE adapter), automatic stacking unit (StackUnit, STONEMASTER UG, Linkenheim-Hochstetten, Germany), and LED (LED-Segment SN1; STONEMASTER UG, Linkenheim-Hochstetten, Germany). The images were stacked using image stacking software (Helicon Pro 7.5.4, HeliconSoft, Kharkiv, Ukraine; method C, smoothing: 2) [40].

For a detailed image of the surface, a confocal laser scanning microscope (VK-X1000/X1050, Keyence, Osaka, Japan; detector resolution: 2048 × 1536 px) and a 100x objective lens (CF IC EPI Plan SLWD 100×, N = 0.73, WD = 4.7 mm) were used to obtain optical images of the surface, which were then converted into monochrome images without color information. Furthermore, differential interference images were created with a red laser (λ = 661 nm) using a 150× lens (CF IC EPI Plan Apo 150x, N = 0.95, WD = 0.2 mm, Nikon, Tokio, Japan).

For quantitative surface texture analysis, 20 surfaces were examined at each time point (baseline, t_(1)_, t_(2)_, t_(3)_) for each material and medium using confocal laser scanning microscopy (VK-X1000/X1050, Keyence, Osaka, Japan; λ = 661 nm; detector resolution: 2048 × 1536 px) at 50× magnification (CF IC EPI Plan 50x, N = 0.8; WD = 0.54 mm; Nikon, Tokio, Japan). The surfaces were first analyzed (MultiFileAnalyzer 2.1.3.89; Keyence Cooperation, Osaka, Japan) according to ISO 25178 for the large-scale surface features via the S-F surface (F-operator: 0.05 mm; S-filter: 0. 8 µm; filter-type: Double Gaussian, end effect correction) and then via the S-L surface (F-operator: 0.05 mm; S-filter: 0.8 µm; L-filter: 0.025 mm; filter-type: Double Gaussian, end effect correction). The following surface parameters were collected for both scale-limited surfaces for all time points (baseline, t_(1)_, t_(2)_, t_(3)_): Sa/µm (arithmetical mean height); Sal/µm (autocorrelation length); Sdr/% (developed interface ratio); Sku (kurtosis); and Ssk (skewness) (see Figure 1) [35]. For a simpler understanding of the results, the S-F surface was partly labeled as “Waviness” and the S-L surface partly as “Roughness”, which is not in line with the formulations in the ISO 25178. The collected parameters Sa, Sal, Sdr, Sku, and Ssk were processed using SPSS 29.0.0.0. A Shapiro–Wilk test for normal distribution, a three-way ANOVA, and Tukey-HSD post hoc multiple comparisons were carried out. The significance level was set at 0.05. To evaluate the sample size, effect sizes were determined according to partial eta squared (*pη*^2^) and Cohen’s *f* [41]. On this basis, a post hoc computed achieved power analysis was performed using G*Power Version 3.1.9.7 with the specified significance level of α = 0.05 [42].

## 3. Results

### 3.1. Mechanical Characterization

The mechanical characterization of the materials showed that JUV had the highest Vickers and Martens hardness, the highest indentation modulus, the lowest creep deformation (under identical load), and the lowest plastic part of indentation work (W_plast_) (Table 2). In contrast, BRE showed the lowest Vickers and Martens hardness values as well as the lowest indentation modulus. VPR showed the second highest hardness (Vickers, Martens), indentation modulus, second lowest creep deformation, and second plastic mechanical work content. VIO and IVO showed similar mechanical properties that were located between BRE and JUV. The differences in Vickers hardness between the materials were smaller than in Martens hardness.

### 3.2. Surface Texture Analysis

**Figure 2:**
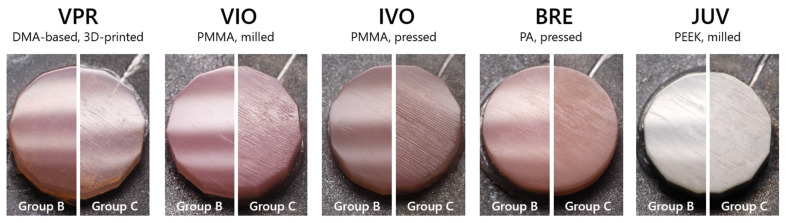
Representative images of the tested materials (Ø: 12 mm) after exposure to a total of 7200 cycles of toothbrushing simulation for groups B (dish detergent) and C (toothpaste slurry).

After the exposure to toothbrushing simulation, similar results were found for groups A and B, both visually and via 3D surface texture analysis, in the resulting surfaces. Therefore, only groups B and C are compared in the following. Mean and median values as well as standard deviation, standard error, and the 95% confidence interval for all groups and all measurement times can be found in the Supplemental Materials. A total of 7200 cycles resulted in differently pronounced surfaces of the tested samples. While only minor scratches could be identified on the sample surface within groups A and B, the samples from group C showed considerably more roughening with loss of gloss, especially for VIO, IVO, BRE, and JUV (Figure 2). VPR showed the least change in the visually assessable surface.

**Figure 3:**
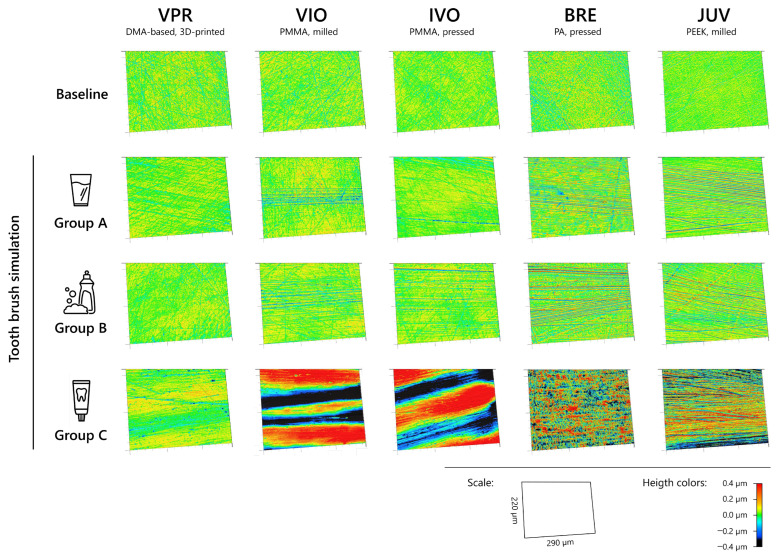
3D surface rendering of analyzed denture base materials before (baseline) and after (t_(3)_) loading via toothbrushing simulation.

**Figure 4:**
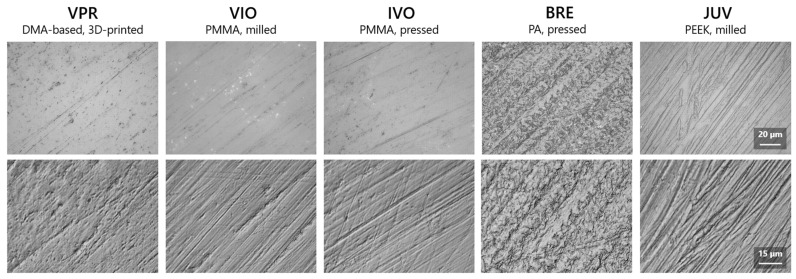
Aged samples from group C (t_(3)_, toothpaste slurry); top: optical images with 100× objective (without color); bottom: differential interference images from laser measurement with 150× objective.

The 3D surface renderings of the examined sample groups confirmed the impressions of the visual assessment (Figure 3). Accordingly, differently pronounced surfaces were observed, especially in group C. While VIO and IVO showed very large valleys and a greater overall surface height, BRE and JUV mainly showed very fine scratch marks (Figure 3). Based on the detailed images (Figure 4), scratches due to abrasive forces were visible on VIO, IVO, and JUV, while BRE exhibited a plastic deformation of the surface without clear scratch lines. VPR showed slight scratch marks. An individual detailed display of the surface renderings for each material can be found in the Supplemental Materials (Figures S1–S5).

The three-factor ANOVA carried out for the surface parameters Sa, Sal, and Sdr for both scale-limited surfaces S-L and S-F showed significant values (*p* < 0.05) for all three factors, material, medium, and time (Table 3). Some significant values (*p* < 0.05) were also determined for Ssk, although the respective F-values were far below those of the before-named parameters. For the parameters Sa, Sal, and Sdr, the highest F-values can be assigned to the factor medium (water/dish detergent/toothpaste slurry). Furthermore, the material (VPR/VIO/IVO/BRE/JUV) is a decisive factor.

Power analysis revealed validity for the effects of material, medium, and time on Sa, Sal, and Sdr (<0.99, see Appendix A, Table A2). For the parameters Sku and Ssk, values between 0.21 and 0.98 were obtained.

**Figure 5:**
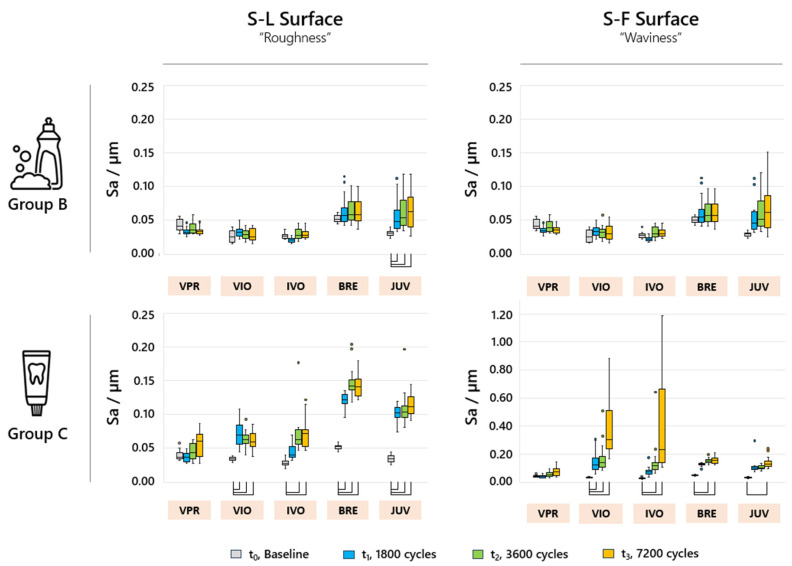
Sa parameters of the analyzed sample groups for the different filter variants S-L and S-F for the analyzed time periods t_(0)_, t_(1)_, t_(2)_, and t_(3)_; the box plots marked with brackets indicate significant differences (*p* < 0.05).

The filtering of the surfaces used resulted in widely differing values for the analyzed parameters. For Sa, the diagrams within group B show similar values for the S-F and S-L surfaces (Figure 5). For group C, VIO and IVO showed similar, large values for Sa, with an increasing trend based on the S-F surface. After filtering the large-scale features for the S-L surface, BRE and JUV showed the highest determined Sa values already after the first measuring point (1800 cycles). VPR showed no significant increase in Sa values for all the analyzed groups and time points.

**Figure 6:**
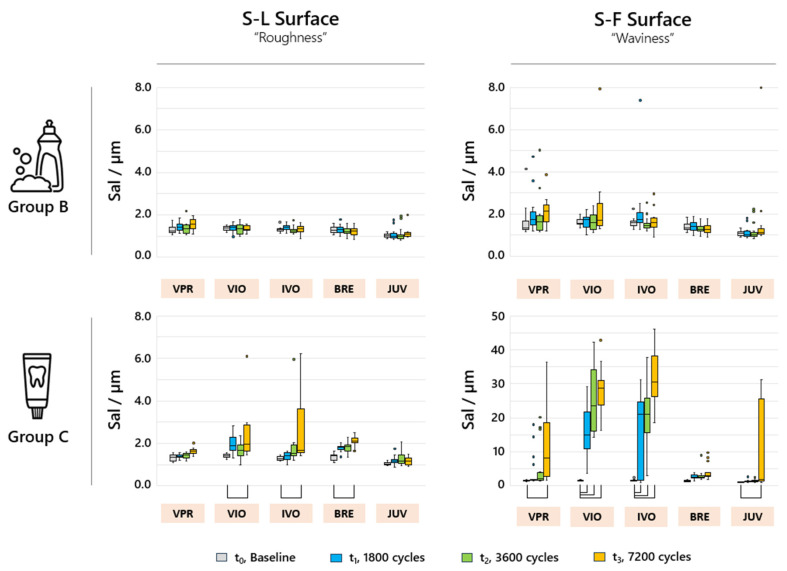
Sal parameters of the analyzed sample groups for the different filter variants S-L and S-F for the analyzed time periods t_(0)_, t_(1)_, t_(2)_, and t_(3)_; the box plots marked with brackets indicate significant differences (*p* < 0.05).

**Figure 7:**
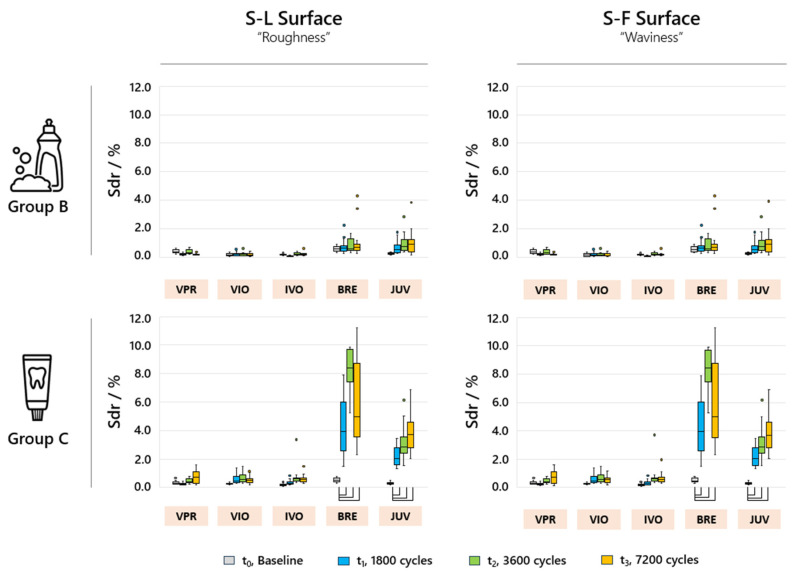
Sdr parameters of the analyzed sample groups for the different filter variants S-L and S-F for the analyzed time periods t_(0)_, t_(1)_, t_(2)_, and t_(3)_; the box plots marked with brackets indicate significant differences (*p* < 0.05).

The analyzed Sal values only showed significant changes (*p* < 0.05) over time for group C (Figure 6). Accordingly, for VIO and IVO, the S-F surface exhibited large Sal values of up to 40 µm for Group C. For BRE and JUV, the Sal values for the S-L surface showed very small intervals of 1–2 µm. After a total of 7200 cycles of toothbrushing simulation, an increase in the specific surface area of 2–12% for BRE and 2–7% for JUV could be demonstrated. No significant increase in the Sdr values was observed for VPR, VIO and IVO. No significant changes were observed in the Sdr parameters for any of the materials or sample groups due to the S-F surface being filtered into the S-L surface (Figure 7).

An overview of all the analyzed sample groups (water, dish detergent, toothpaste slurry) and collected parameters (Sa, Sal, Sdr, Sku, Ssk) can be found in the Supplemental Materials (Figures S5–S10).

## 4. Discussion

### 4.1. Mechanical Characteristics

During the toothbrushing simulation, a vertical force was applied on the material surfaces via the contact pressure (2.5 N~250 g distributed over the brush surface), and an additional horizontal force was applied due to the brush movement. In addition, frictional forces occurred during the movement, depending on the medium used. The pronounced differences between the loads with non-abrasive (A, B) and abrasive (C) media suggest that the frictional forces are only significantly influenced (*p* < 0.05) by abrasive components. In contrast to Martens hardness (HM), Vickers hardness (HV) only takes the plastic, non-reversible deformation into account, which is associated with a change in surface texture. Therefore, Vickers hardness probably has a greater impact in terms of abrasion resistance compared to Martens hardness. Although Momber et al. (2020) demonstrated a significant influence of Vickers hardness on the abrasion resistance of polymer materials, they also noted that there are further mechanical characteristics that are relevant for surface stability [43]. Even if the plastic range is more relevant in this regard, a high modulus of elasticity indicates a generally low deformability under identical loads. This low deformability results in higher horizontal forces even with an identical texture. Softer, more flexible materials with a lower modulus of elasticity can therefore better deform back than more brittle materials with a high modulus of elasticity [44]. This effect could be amplified if the specific surface area is reduced by the elastic deformation, which would result in lower stresses as acting force in relation to surface area. Finally, it should be noted that abrasion was not quantified in this study. Materials with a low abrasion resistance could therefore not necessarily exhibit a strongly altered surface texture. Regarding the affinity to biofilm, however, surface abrasion plays a subordinate role in contrast to texture.

The Vickers and Martens hardness determined were confirmed in the ranking between the products examined by the literature data [12,19,20,45,46,47]. The highest Vickers/Martens hardness was found for PEEK, followed by the DMA-based and PMMA polymers. No hardness but comparatively low flexural strengths were found in the literature for PA-based denture base materials [45]. Contrary to expectations, both with water and dish detergent, but above all with abrasive cleaning, PA with the lowest and PEEK with the highest Vickers hardness showed almost comparable changes in surface texture. A direct dependency between Vickers hardness and individual surface texture parameters could therefore not be derived. Due to the analyzed high E-modulus (E_IT_) and very low plastic compared to elastic indentation work (W_elast_ and W_plast_) as well as the low creep behavior (C_IT_), a comparatively low deformation fraction could be detected in PEEK. This results in higher local stresses in the microstructure under load and thus a brittle failure close to the surface, which presumably results in erosion. At the same time, PA with lower structural integrity also showed significant changes (*p* < 0.05) in the surface texture. PA, also known as nylon, is known for its very high toughness (ductility), which was demonstrated by its very high plastic (W_plast_) compared to elastic (W_elast_) part of indentation work.

In contrast to brittle materials, the ductile behavior of PA enables force absorption through plastic, non-reversible deformation even when its strength (defined as the maximum stress) is exceeded [48]. The surface images of PA showed corresponding ductile deformation on the surface, while PEEK exhibited scratch marks. Consequently, the texture changes in PEEK can be explained by an erosion and in PA by a reversible deformation. Further investigations would be necessary, in which not only texture parameters but also local thickness changes are measured.

### 4.2. Surface Texture

The results provide a comprehensive overview of the denture base materials after artificial ageing. The mode of loading, either abrasive or non-abrasive, is crucial, followed by the choice of denture base material for an increased surface roughness. In addition, the parameters Sa, Sal, and Sdr provided a good description of the surface changes, whereas Sku and Ssk proved to be ineffective parameters for analysis of these surface changes. The power analysis performed showed a sufficient number of analyzed areas (*n* = 20 per group) for Sa, Sal, and Sdr and, in some cases, for Sku and Ssk. The use of water and dish detergent did not result in any major changes in the surface texture. It can be assumed that the chemical effect of the detergents (e.g., in denture cleaning foam) does not contribute to any particular increase in surface roughness. Only for PEEK, a statistically significant (*p* < 0.05) but small increase in Sa from 0.03 to 0.05 µm occurred after the first loading time of 1800 cycles (equivalent to 3 months). For abrasive cleaning, the different filtering of the surfaces showed more detailed results. The highest Sa values were found for both PMMA-based denture base materials via the S-F surface (“waviness”), while the greatest surface changes for the PA- and PEEK-based materials were observed via the S-L surface (“roughness”). Consequently, the short-scale surface features based on small scratches characterize the surfaces for PA and PEEK. Although, wide valleys, as with PMMA, have no influence on the available total surface area. For the DMA-based printed material, the smallest changes were generally observed after toothbrushing simulation, so that a higher resistance to biofilm deposition in clinical use could be potentially expected. This observation is very promising, as 3D-printed denture base material has previously been shown to have higher microbial adhesion than heat-pressed or milled PMMA [49,50,51]. Nonetheless, artificial ageing was not sufficiently taken into account, as it should be urgently considered in clinical implementation. Especially here, the long-term stability of the materials and biofilm development should be seen in context.

For clinical classification and the formulation of important threshold values for biofilm deposition and development, the literature often refers to an established threshold of Ra < 0.2 µm according to Bollen et al. (1996) using titanium and ceramic abutments [52]. Even though attempts have been made to extend the threshold value to dental polymers, since the establishment of areal 3D surface texture analysis, the methods are no longer up-to-date or in some cases insufficiently methodologically documented due to a lack of information on magnification or filter settings and are therefore difficult to compare [53,54,55]. Since the determined Sa value reduces with a higher lens magnification [56], it can be assumed that the arithmetical mean heights of PA and PEEK documented by this study of 0.10 to 0.15 µm are below the established threshold of 0.20 µm but still represent a potential risk for increased biofilm deposition and development. In addition, without appropriate filtering into the S-L surface, the largest mean arithmetic height would have resulted for PMMA. At this point, further studies on the comparison of current surface parameters with biofilm development and detailed information on the methodology used (objective, resolution, laser wavelength, filter settings) are urgently recommended.

### 4.3. Clinical Implications

The early stages of biofilm formation play a key role in the attachment of pathogenic bacteria and fungi and the resulting oral diseases, especially if niches in the micrometer range are available for good adhesion and subsequent biofilm growth and protect early colonizers from shear forces [57,58]. The surface-dominating valleys/niches of the PA- and PEEK-based polymers are located at 1–2 µm in a dimension of relevant bacteria such as Candida albicans, Streptococcus oralis, Streptococcus gordonii, or Fusobacterium nucleatum and should therefore be considered as a particularly favorable factor for biofilm development [57,58,59,60]. In addition, the analysis of surface texture via Sdr clearly shows that both the S-F and the resulting S-L surface area by filtration had almost identical specific surface area fractions for all analyzed materials. The developed size of the total specific surface area was determined by the small surface features. The relationship between PMMA of both manufacturing processes and PA and PEEK shows that a high Sa value is not a suitable sole indicator for the specific surface area and that this should be considered separately by the Sdr. The available area for potential biofilm deposition was already maximized for PEEK, but especially for PA, after three months of abrasive cleaning. In clinical use, polishing of PA- or PEEK-based denture materials may be warranted after three months of abrasive cleaning.

PEEK is used because of its increased mechanical stability and thus higher wear resistance. [16,61]. Based on these findings, the mechanical resistance of PEEK should be examined more closely in similar study designs.

### 4.4. Limitations

A separate polishing regime is available for PA, which was not considered in this study, but should be taken into account in clinical use. Additionally, the investigation of the materials is linked to specific manufacturing processes. To enable separate evaluation of the individual factors—material, manufacturing process, and artificial aging—additional tests should be conducted using a broader selection of materials. Moreover, relevant mechanical characteristics such as breaking strength or bending tensile strength were not investigated. Other clinical influences should also be considered, as this study was only focused on simulated cleaning. Thermocycling, water storage, and other ageing protocols are therefore important procedures that can influence the properties of denture materials and provide an improved assessment of long-term material behavior.

Finally, the authors would like to point out that the observed 3D surface texture parameters were selected to assess surface height, distribution of the surface, size of the available surface, and texture size. These parameters were considered suitable for evaluating biofilm formation and development in terms of bacterial attachment. Due to the development of areal surface measurements, studies are urgently needed to validate these parameters under a comparable analysis setup (e.g., 50× magnification and appropriate filtering for small surface features) and estimate the correlation.

## 5. Conclusions

Knowledge of the material is important, as the manufacturing process determines the choice of material and thus indirectly the long-term behavior. A comprehensive analysis according to ISO 25178 with suitable filtering provides a good and detailed reproduction of the surface structure, according to which the resistance of the denture base materials to cleaning processes can be described as follows:

- Regardless of the choice of material, water and dish detergent cause negligible changes to the surface.
- Printed DMA-based denture base materials show no relevant damage due to abrasive cleaning.
- Both milled and pressed PMMA show considerable changes after abrasive cleaning, which can be attributed to coarse surface features. These changes are not considered critical for biofilm formation.
- PA and PEEK exhibit rough surfaces with a large specific surface area for potential biofilm adhesion after abrasive cleaning.

## Figures and Tables

**Table 1:** Overview of the materials used with the abbreviation used and the information according to the manufacturer information with UDMA (urethane dimethacrylate), TEGDMA (Triethylenglycoldimethacrylat), PMMA (polymethyl metacrylate), PA (polyamide), and PEEK (polyether ether ketone).

Code	Material	Preparation	Composition	Name	Manufacturer	LOT
VPR	DMA-based	3D-printed	50–75 wt% aliphatic UDMA 2.5–10 wt% TEGDMA 0.1–1 wt% photoinitiator	V-Print dentbase	VOCO GmbH	2143120
VIO	PMMA	milled	99 wt% PMMA 1 wt% pigments	Vita Vionic Base	VITA Zahnfabrik	76380
IVO	PMMA	pressed	>95 wt% PMMA <2.5 wt% dibenzoyl peroxide	IvoBase Hybrid	Ivoclar Vivadent	YB38C7
BRE	PA	pressed	>99.9 wt% PA <0.1 wt% pigments	Bre.flex 2nd Edition	bredent	383425
JUV	PEEK	milled	100% PEEK	Juvora Disc	JUVORA	M000910

**Table 2:** Mean and standard deviation for Martens hardness (HM), indentation modulus (E_IT_), elastic and plastic part of indentation work (W_elast_, W_plast_), indentation creep (C_IT_), and Vickers hardness (HV1) of the materials.

Group	HM	E_IT_	C_IT_	W_elast_	W_plast_	HV1
	MPa	kN/mm^2^	%	Nmm	Nmm	-
VPR	182.3 ± 0.9	4.4 ± 0.1	6.9 ± 0.2	0.074 ± 0.001	0.104 ± 0.001	26.5 ± 1.7
VIO	165.4 ± 0.8	4.4 ± 0.1	9.6 ± 0.2	0.066 ± 0.001	0.130 ± 0.001	21.9 ± 0.4
IVO	185.2 ± 3.5	4.2 ± 0.2	9.2 ± 0.6	0.068 ± 0.001	0.135 ± 0.001	22.2 ± 0.6
BRE	67.1 ± 1.6	1.7 ± 0.1	5.8 ± 0.3	0.112 ± 0.001	0.200 ± 0.001	9.0 ± 0.1
JUV	220.1 ± 2.5	5.3 ± 0.1	3.5 ± 0.1	0.066 ± 0.001	0.084 ± 0.001	27.9 ± 0.9

**Table 3:** Three-factor ANOVA for 3D surface texture parameters for each S-L and S-F surface filterings; combined factors for material, medium, and time can be found in the Appendix A (Table A1).

	Sa		Sal		Sdr		Sku		Ssk	
	*F*	*p*-Value	*F*	*p*-Value	*F*	*p*-Value	*F*	*p*-Value	*F*	*p*-Value
**S-L surface (Roughness)**										
Material	441.3	<0.001	41.1	<0.001	207.0	<0.001	1.7	0.159	2.3	0.059
Medium	609.5	<0.001	68.6	<0.001	284.8	<0.001	1.8	0.170	1.6	0.209
Time	148.7	<0.001	22.0	<0.001	60.7	<0.001	0.7	0.523	4.6	0.003
**S-F surface (Waviness)**										
Material	12.7	<0.001	116.3	<0.001	205.1	<0.001	1.7	0.143	5.4	<0.001
Medium	187.2	<0.001	514.9	<0.001	287.1	<0.001	1.9	0.145	1.5	0.216
Time	57.3	<0.001	106.1	<0.001	61.2	<0.001	0.7	0.538	4.0	0.007

## Data Availability

The raw data supporting the conclusions of this article will be made available by the authors on reasonable request.

## Supplementary Materials

The following supporting information can be downloaded at: https://www.mdpi.com/article/10.3390/jfb16100359/s1, Table S1. Mean, median, standard deviation (SD), standard error (SE) and 95%-confidence interval for the Sa parameters of the analyzed sample groups for the different filter variants S-L and S-F for the analyzed time periods t0, t1, t2 and t3. Table S2. Mean, median, standard deviation (SD), standard error (SE) and 95%-confidence interval for the Sal parameters of the analyzed sample groups for the different filter variants S-L and S-F for the analyzed time periods t0, t1, t2 and t3. Table S3. Mean, median, standard deviation (SD), standard error (SE) and 95%-confidence interval for the Sdr parameters of the analyzed sample groups for the different filter variants S-L and S-F for the analyzed time periods t0, t1, t2 and t3. Table S4. Mean, median, standard deviation (SD), standard error (SE) and 95%-confidence interval for the Sku parameters of the analyzed sample groups for the different filter variants S-L and S-F for the analyzed time periods t0, t1, t2 and t3. Table S5. Mean, median, standard deviation (SD), standard error (SE) and 95%-confidence interval for the Ssk parameters of the analyzed sample groups for the different filter variants S-L and S-F for the analyzed time periods t0, t1, t2 and t3. Figure S1. VPR for the baseline and after exposure to the toothbrush simulation (t(3), 7200 cycles in total) with the water (Group A), dish detergent (Group B) or toothpaste slurry (Group C). Figure S2. VIO for the baseline and after exposure to the toothbrush simulation (t(3), 7200 cycles in total) with the water (Group A), dish detergent (Group B) or toothpaste slurry (Group C). Figure S3. IVO for the baseline and after exposure to the toothbrush simulation (t(3), 7200 cycles in total) with the water (Group A), dish detergent (Group B) or toothpaste slurry (Group C). Figure S4. BRE for the baseline and after exposure to the toothbrush simulation (t(3), 7200 cycles in total) with the water (Group A), dish detergent (Group B) or toothpaste slurry (Group C). Figure S5. JUV for the baseline and after exposure to the toothbrush simulation (t(3), 7200 cycles in total) with the water (Group A), dish detergent (Group B) or toothpaste slurry (Group C). Figure S6. Sa parameters of the analyzed sample groups for the different filter variants S-L and S-F for the analyzed time periods t(0) (grey), t(1) (blue), t(2) (green), and t(3) (yellow). Figure S7. Sal parameters of the analyzed sample groups for the different filter variants S-L and S-F for the analyzed time periods t(0) (grey), t(1) (blue), t(2) (green), and t(3) (yellow). Figure S8. Sdr parameters of the analyzed sample groups for the different filter variants S-L and S-F for the analyzed time periods t(0) (grey), t(1) (blue), t(2) (green), and t(3) (yellow). Figure S9. Sku parameters of the analyzed sample groups for the different filter variants S-L and S-F for the analyzed time periods t(0) (grey), t(1) (blue), t(2) (green), and t(3) (yellow). Figure S10. Ssk parameters of the analyzed sample groups for the different filter variants S-L and S-F for the analyzed time periods t(0) (grey), t(1) (blue), t(2) (green), and t(3) (yellow).

## Abbreviations

The following abbreviations are used in this manuscript:

DMA	dimethyl methacrylate
PMMA	polymethyl methacrylate
PA	polyamide
PEEK	polyether ether ketone
VPR	V-Print dentbase
VIO	VITA Vionic Base
IVO	IvoBase Hybrid
BRE	Bre.flex
JUV	Juvora Disc
VH	Vickers hardness
HM	Martens hardness
E_IT_	indentation modulus
W_elast_	elastic plastic part of indentation work
W_plast_	plastic part of indentation work
C_IT_	indentation creep
Sa	arithmetical mean height
Sal	autocorrelation length
Sdr	developed interface ratio
Sku	kurtosis
Ssk	skewness

## Appendix A

**Table A1:** Results of the three-way ANOVA for the surface texture parameters Sa. Sal, Sdr, Sku, and Ssk for the different filter variants S-L and S-F surface.

	Sa		Sal		Sdr		Sku		Ssk	
	F	*p*-Value	F	*p*-Value	F	*p*-Value	F	*p*-Value	F	*p*-Value
**S-L surface (‘Roughness’)**										
Material	441.3	<0.001	41.1	<0.001	207.0	<0.001	1.7	0.159	2.3	0.059
Medium	609.5	<0.001	68.6	<0.001	284.8	<0.001	1.8	0.170	1.6	0.209
Time	148.7	<0.001	22.0	<0.001	60.7	<0.001	0.7	0.523	4.6	0.003
Material*Medium	35.0	<0.001	10.5	<0.001	84.8	<0.001	2.0	0.049	4.0	<0.001
Material*Time	25.5	<0.001	2.6	0.002	24.1	<0.001	0.7	0.761	0.6	0.833
Medium*Time	66.0	<0.001	18.9	<0.001	36.3	<0.001	0.7	0.654	1.8	0.088
Material*Medium*Time	7.7	<0.001	4.0	<0.001	14.4	<0.001	0.7	0.855	1.0	0.490
**S-F surface (‘Waviness’)**										
Material	12.7	<0.001	116.3	<0.001	205.1	<0.001	1.7	0.143	5.4	0.000
Medium	187.2	<0.001	514.9	<0.001	287.1	<0.001	1.9	0.145	1.5	0.216
Time	57.3	<0.001	106.1	<0.001	61.2	<0.001	0.7	0.538	4.0	0.007
Material*Medium	19.8	<0.001	90.1	<0.001	83.9	<0.001	2.0	0.049	4.5	<0.001
Material*Time	7.8	<0.001	14.6	<0.001	23.9	<0.001	0.7	0.770	0.6	0.831
Medium*Time	42.4	<0.001	93.7	<0.001	36.5	<0.001	0.7	0.647	1.5	0.164
Material*Medium*Time	8.1	<0.001	13.7	<0.001	14.3	<0.001	0.7	0.869	1.3	0.154

**Table A2:** Effect sizes (with the partial η^2^ and Cohen’s f) and F values from the ANOVA (F). Results of the post hoc computed achieved power analysis for the critical F (F_crit_) and the achieved power (Power) for α = 0.05.

Parameter	Effect	Partial η^2^	Cohen’s f	F	F_crit_	Power
**S-L surface (Roughness)**						
Sa	Material	0.608	1.245	441.28	2.30	>0.999
	Medium	0.517	1.035	609.53	3.00	>0.999
	Time	0.282	0.626	148.68	2.61	>0.999
Sal	Material	0.126	0.380	41.11	2.38	>0.999
	Medium	0.108	0.347	68.61	3.00	>0.999
	Time	0.055	0.241	22.04	2.61	>0.999
Sdr	Material	0.421	0.853	206.95	2.38	>0.999
	Medium	0.334	0.707	284.81	3.00	>0.999
	Time	0.138	0.400	60.72	2.61	>0.999
Sku	Material	0.006	0.076	1.65	2.38	0.511
	Medium	0.003	0.056	1.78	3.00	0.375
	Time	0.002	0.044	0.75	2.61	0.209
Ssk	Material	0.008	0.089	2.28	2.38	0.664
	Medium	0.003	0.052	1.57	3.00	0.329
	Time	0.012	0.111	4.64	2.61	0.897
**S-F surface (Waviness)**						
Sa)	Material	0.043	0.211	12.66	2.30	>0.999
	Medium	0.248	0.574	187.24	3.00	>0.999
	Time	0.131	0.389	57.30	2.61	>0.999
Sal	Material	0.290	0.640	116.27	2.38	>0.999
	Medium	0.475	0.952	514.91	3.00	>0.999
	Time	0.219	0.529	106.12	2.61	>0.999
Sdr	Material	0.419	0.849	205.07	2.38	>0.999
	Medium	0.335	0.710	287.10	3.00	>0.999
	Time	0.139	0.402	61.16	2.61	>0.999
Sku	Material	0.006	0.078	1.72	2.38	0.535
	Medium	0.003	0.058	1.94	3.00	0.400
	Time	0.002	0.044	0.72	2.61	0.209
Ssk	Material	0.019	0.138	5.43	2.38	0.976
	Medium	0.003	0.052	1.54	3.00	0.329
	Time	0.011	0.103	4.03	2.61	0.843
