# Supplementary material for: Effect of Cleaning Protocols on Surface Roughness of Current Polymeric Denture Materials
Source: J Funct Biomater. 2025 Sep 24;16(10):359. doi: 10.3390/jfb16100359 (PMC12565616; doi:10.3390/jfb16100359)
Supplement: Supplementary file 1 [file jfb-16-00359-s001.zip › Supplements_Surface Renderings and Boxplots.pdf]

Journal of
Functional Biomaterials

Supplemental Material

Effect of cleaning protocols on surface roughness of current polymeric denture materials

This supplementary material contains detailed surface renderings (S1-S5) for all tested groups (baseline, water, dish detergent, and toothpaste) at after $t_{(3)}$, (7200 cycles of toothbrushing simulation). Furthermore, all box plot figures for all observed 3D surface texture parameters (S6-S10). All sample groups of the materials VPR, VIO, IVO, BRE, and JUV were considered at all time points $t_{(0)}$ (baseline), $t_{(1)}$ (corresponds to 3 months), $t_{(2)}$ (corresponds to 6 months), and $t_{(3)}$ (corresponds to 12 months) using all media (water, dish detergent, toothpaste slurry). To separate small- and large-scale surface features, the 3D surface texture parameters Sa, Sal, Sdr, Sku, and Ssk were collected for S-L ("roughness") and S-F ("waviness"), respectively.

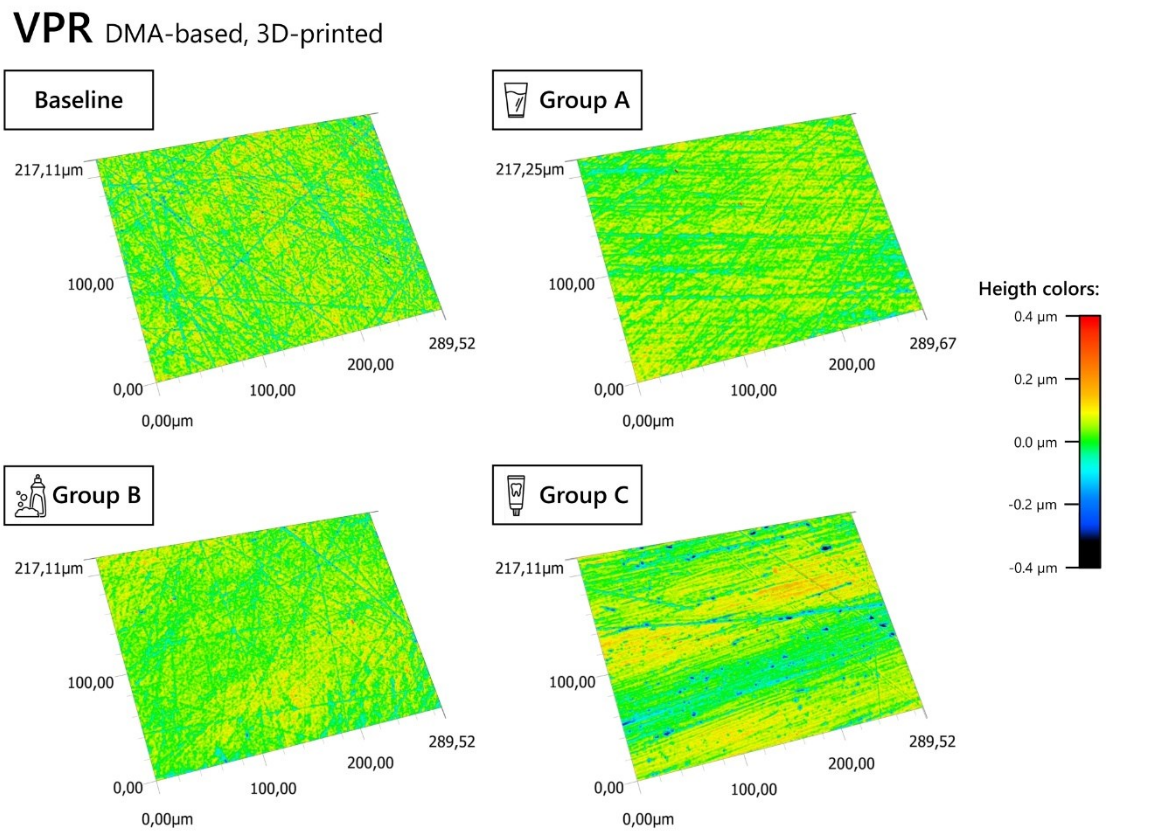

Figure S1. VPR for the baseline and after exposure to the toothbrush simulation ($t_{(3)}$, 7200 cycles in total) with the water (Group A), dish detergent (Group B) or toothpaste slurry (Group C).

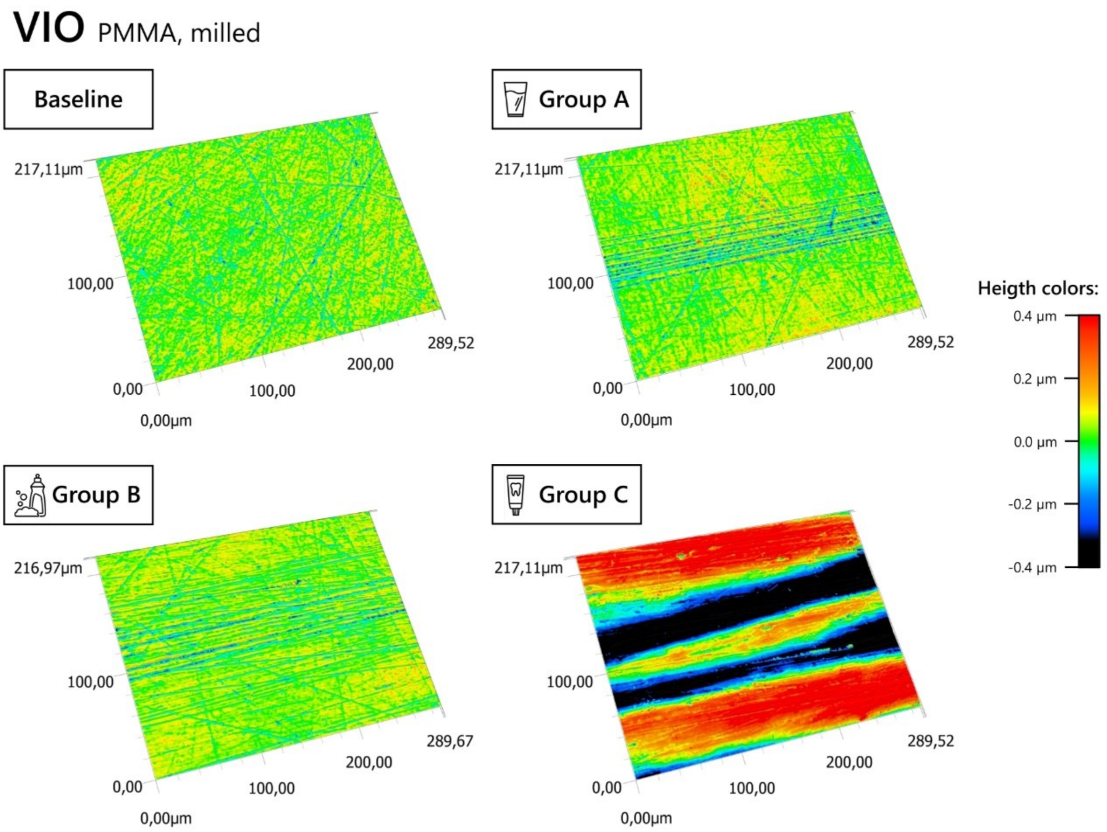

Figure S2. VIO for the baseline and after exposure to the toothbrush simulation ($t_{(3)}$, 7200 cycles in total) with the water (Group A), dish detergent (Group B) or toothpaste slurry (Group C).

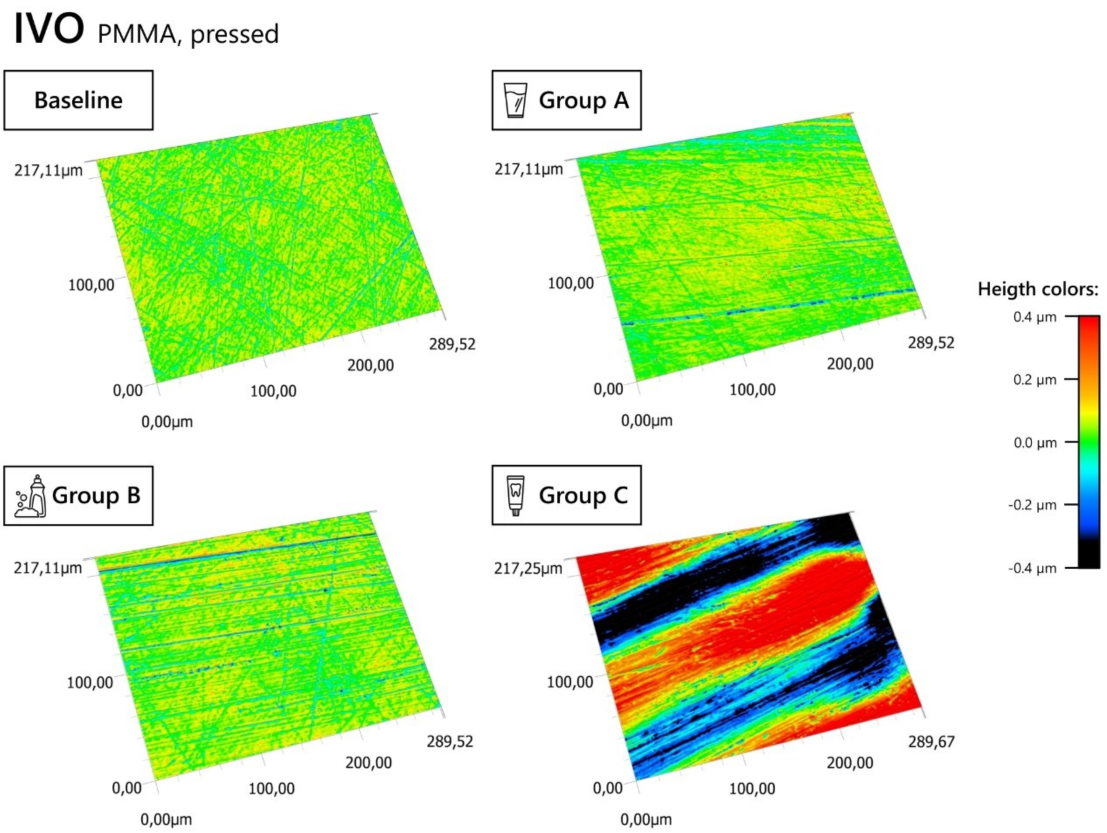

Figure S3. IVO for the baseline and after exposure to the toothbrush simulation ($t_{(3)}$, 7200 cycles in total) with the water (Group A), dish detergent (Group B) or toothpaste slurry (Group C).

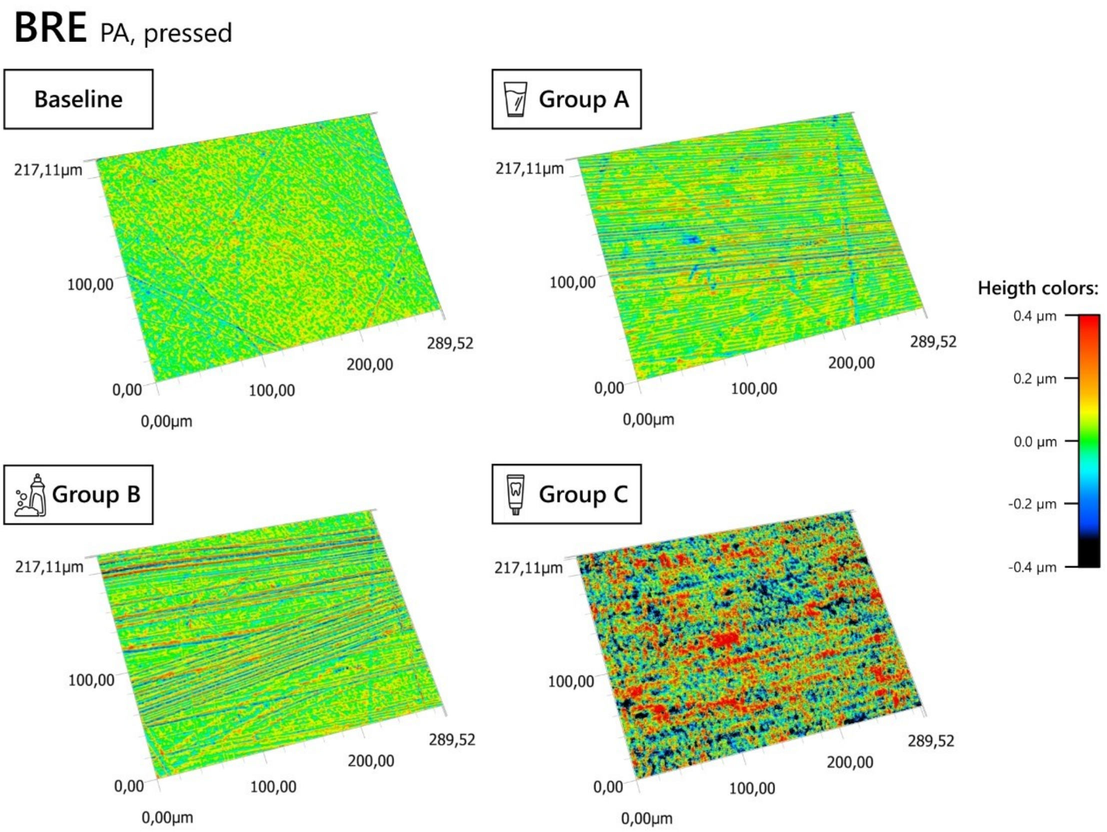

Figure S4. **BRE for the baseline and after exposure to the toothbrush simulation ($t_{(3)}$, 7200 cycles in total) with the water (Group A), dish detergent (Group B) or toothpaste slurry (Group C).**

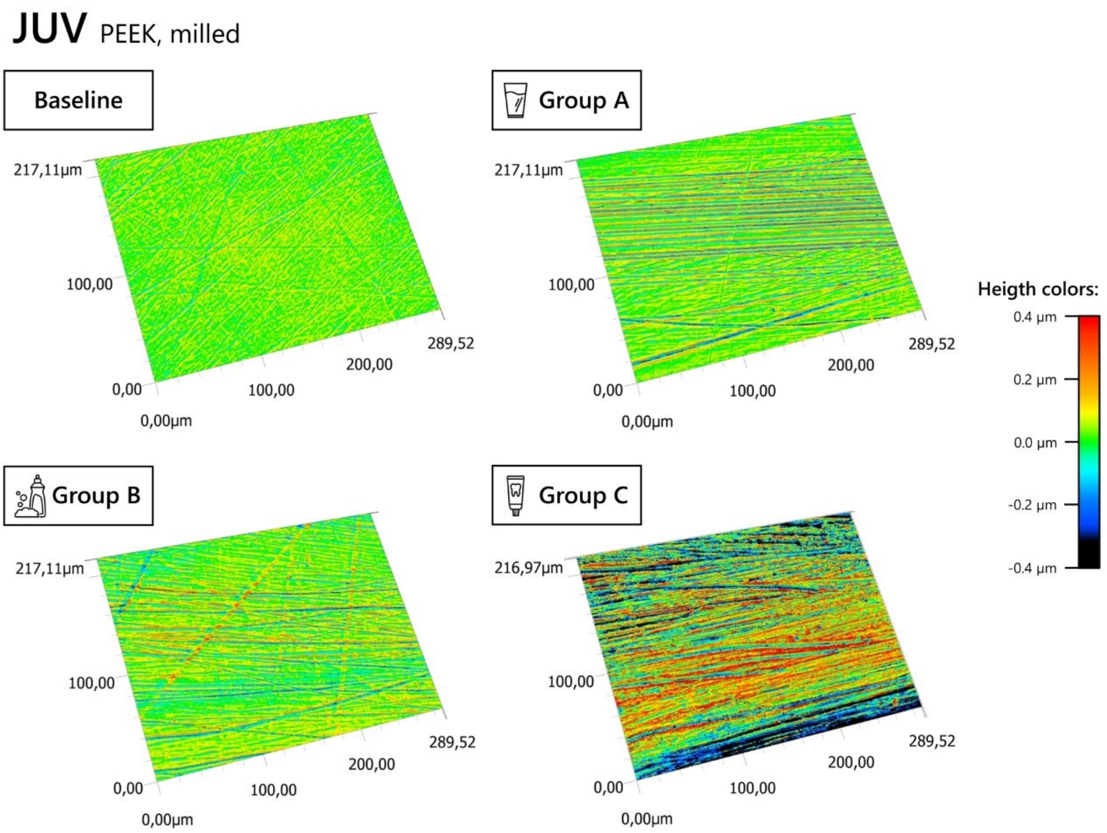

Figure S5. **JUV for the baseline and after exposure to the toothbrush simulation ($t_{(3)}$, 7200 cycles in total) with the water (Group A), dish detergent (Group B) or toothpaste slurry (Group C).**

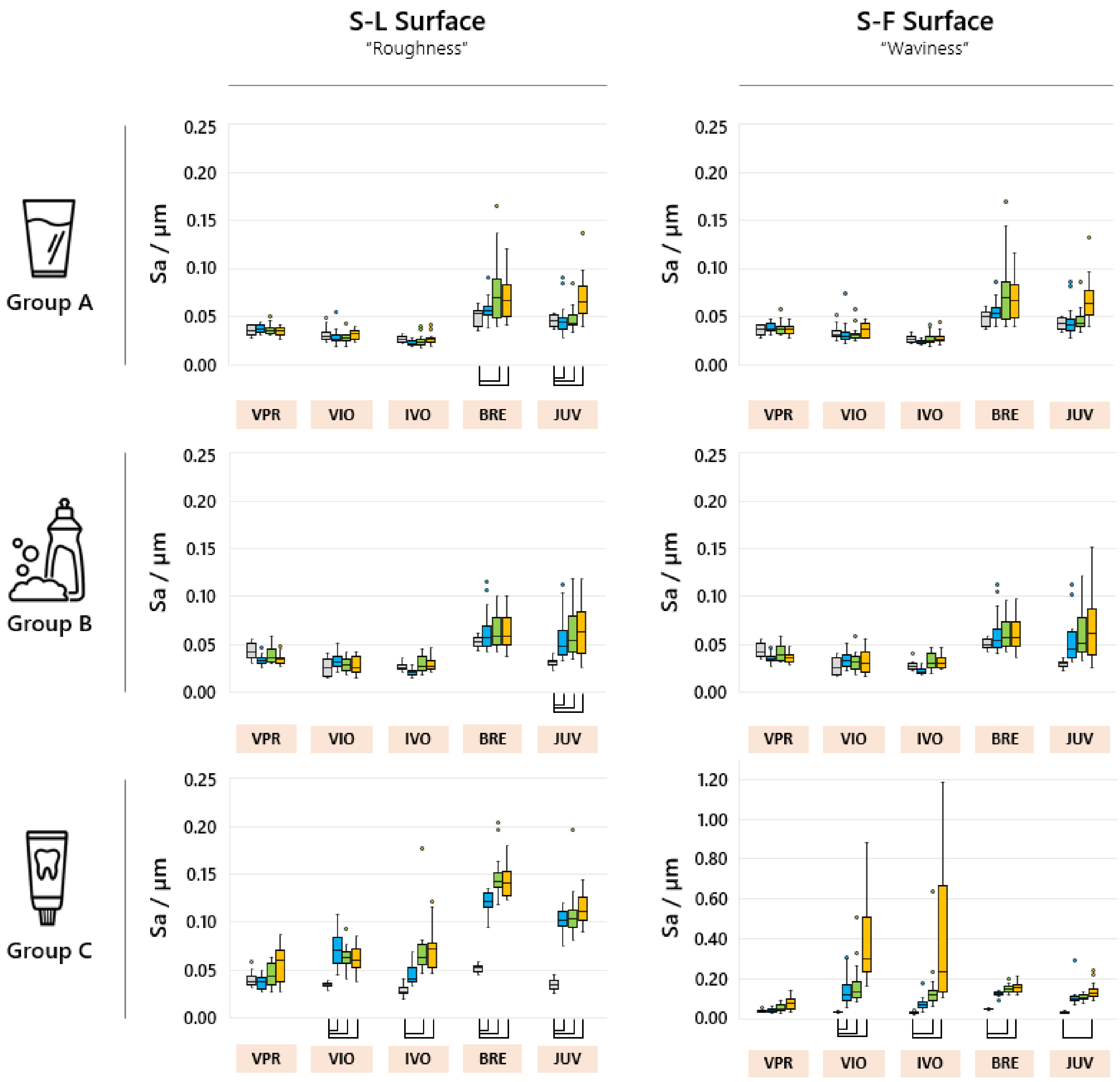

Figure S6. Sa parameters of the analyzed sample groups for the different filter variants S-L and S-F for the analyzed time periods $t_{(0)}$ (grey), $t_{(1)}$ (blue), $t_{(2)}$ (green), and $t_{(3)}$ (yellow).

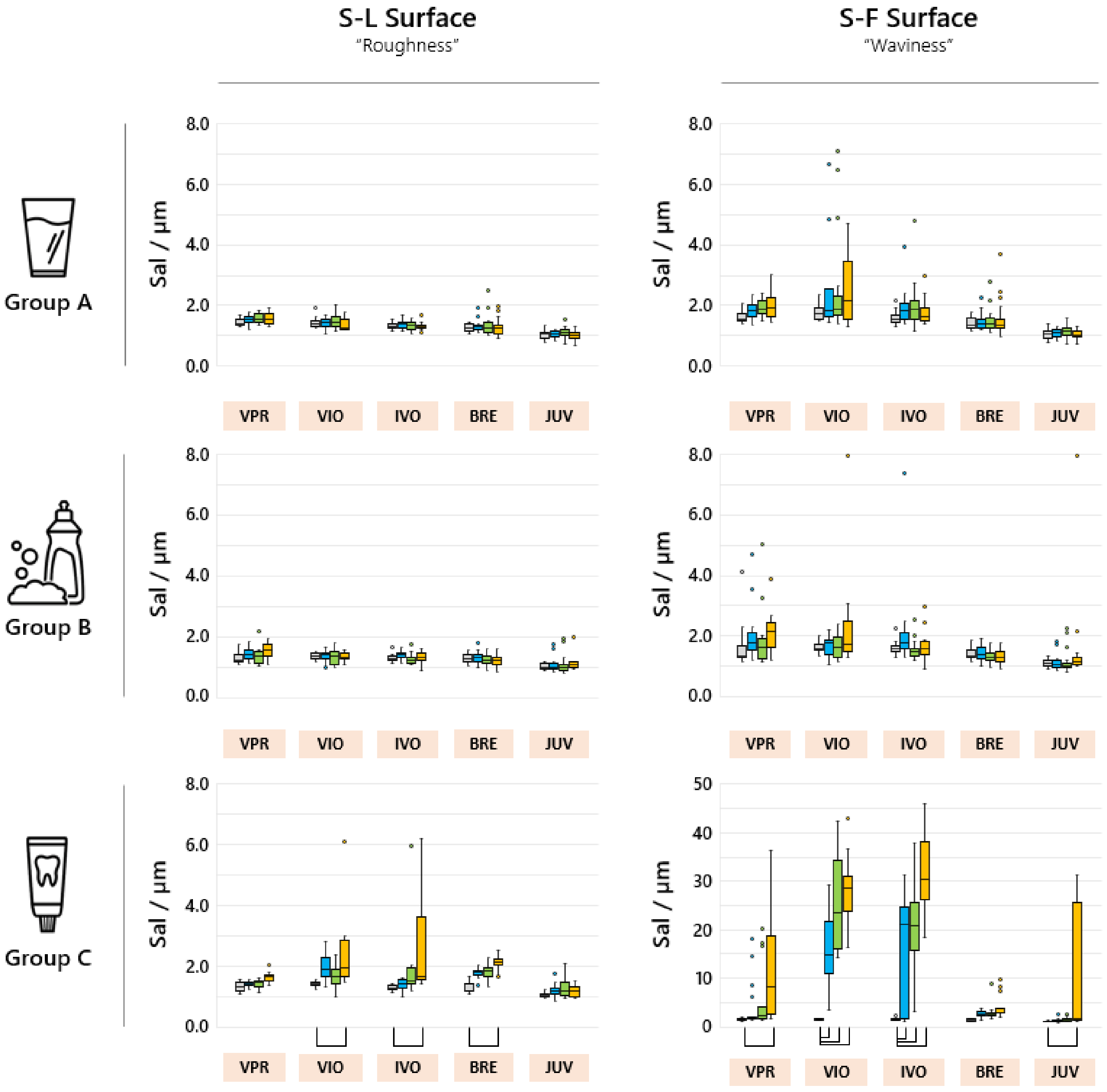

Figure S7. Sal parameters of the analyzed sample groups for the different filter variants S-L and S-F for the analyzed time periods $t_{(0)}$ (grey), $t_{(1)}$ (blue), $t_{(2)}$ (green), and $t_{(3)}$ (yellow).

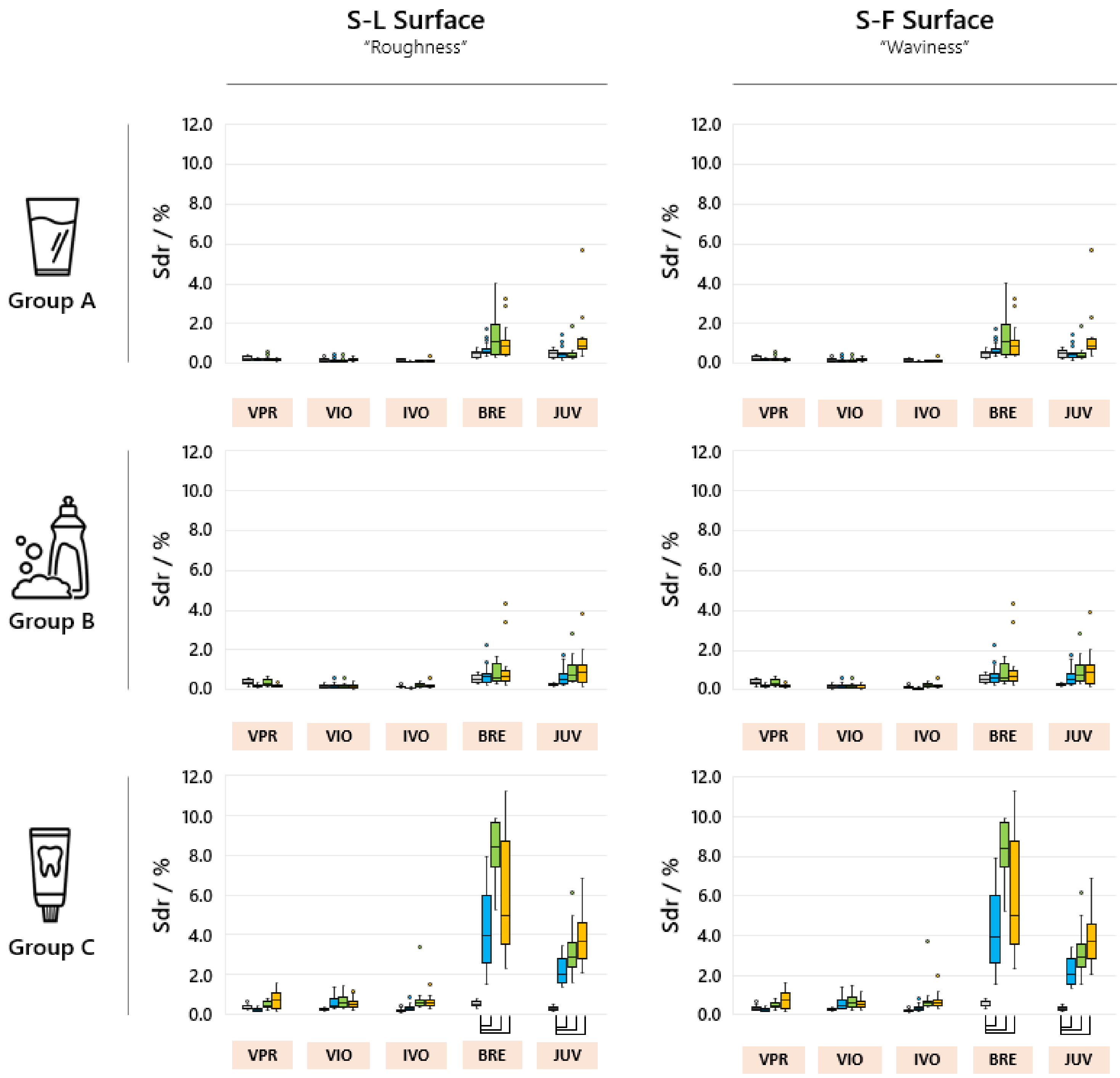

Figure S8. Sdr parameters of the analyzed sample groups for the different filter variants S-L and S-F for the analyzed time periods $t_{(0)}$ (grey), $t_{(1)}$ (blue), $t_{(2)}$ (green), and $t_{(3)}$ (yellow).

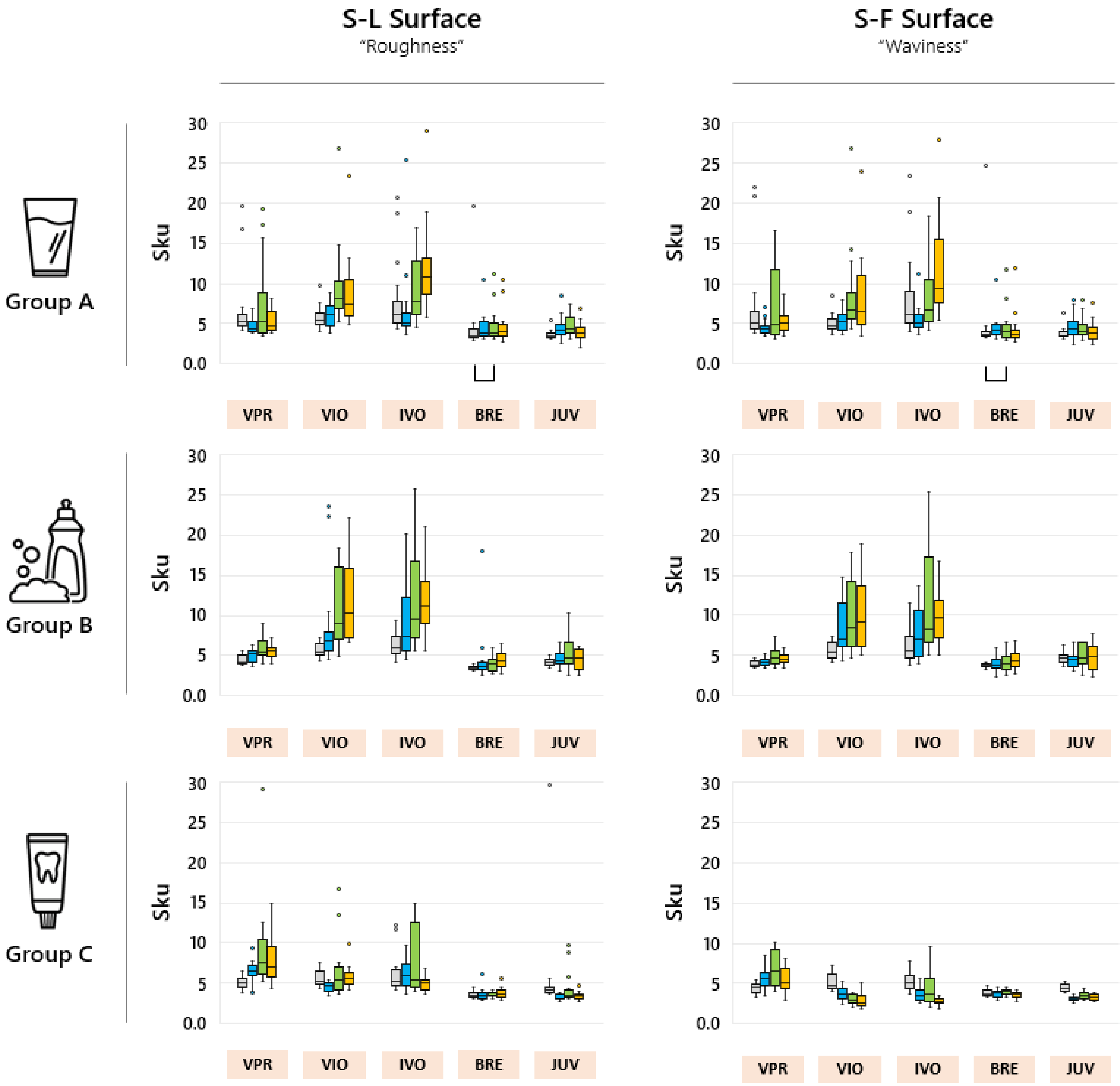

Figure S9. Sku parameters of the analyzed sample groups for the different filter variants S-L and S-F for the analyzed time periods $t_{(0)}$ (grey), $t_{(1)}$ (blue), $t_{(2)}$ (green), and $t_{(3)}$ (yellow).

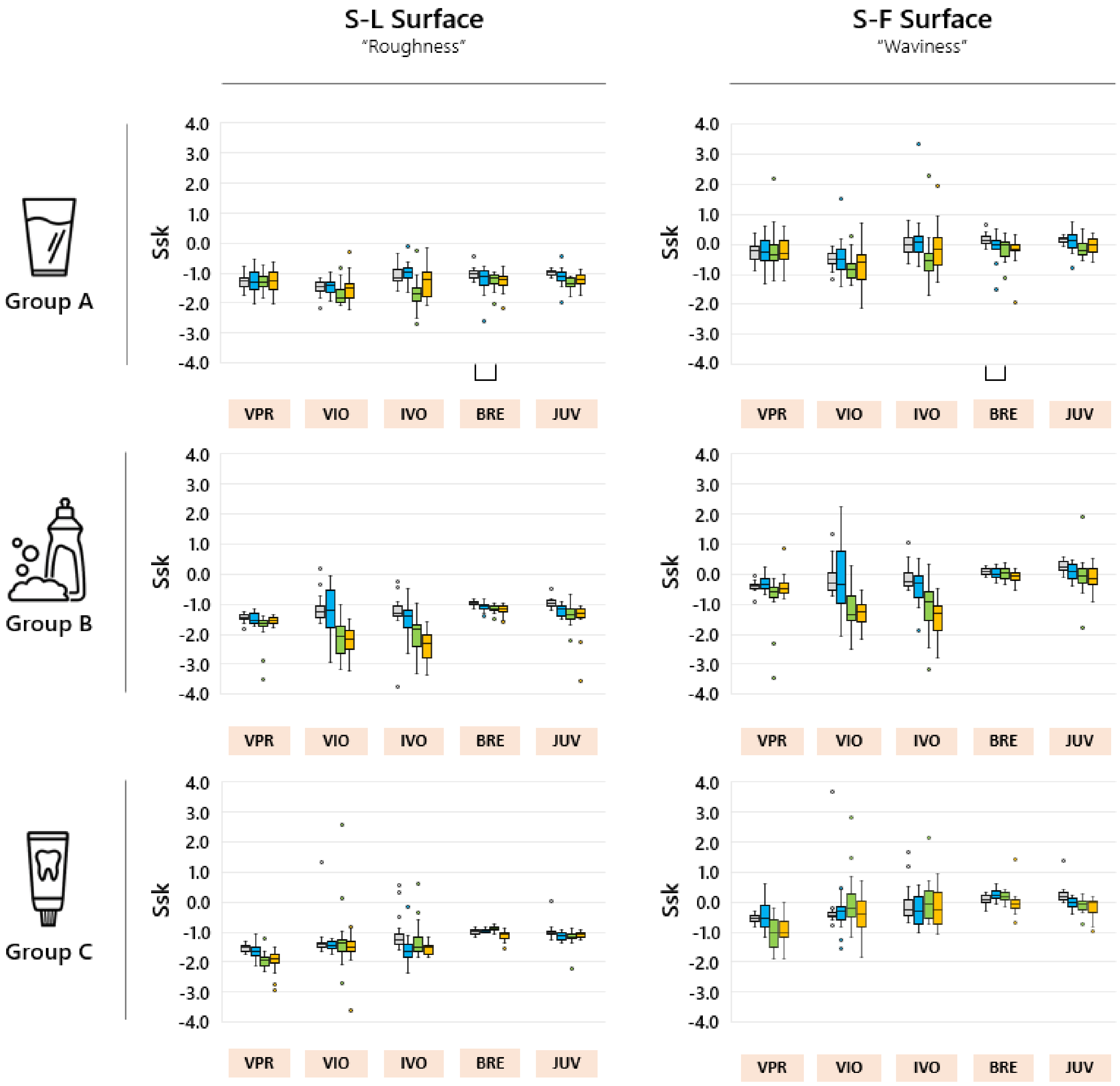

Figure S10. Ssk parameters of the analyzed sample groups for the different filter variants S-L and S-F for the analyzed time periods $t_{(0)}$ (grey), $t_{(1)}$ (blue), $t_{(2)}$ (green), and $t_{(3)}$ (yellow).
